# Embryo–Maternal Interactions Underlying Reproduction in Mammals

**DOI:** 10.3390/ijms21144872

**Published:** 2020-07-10

**Authors:** Stefan Bauersachs, Carmen Almiñana

**Affiliations:** 1 Functional Genomics, Institute of Veterinary Anatomy, Vetsuisse Faculty, University of Zurich, 8315 Lindau (ZH), Switzerland; 2 UMR85 PRC, INRAE, CNRS 7247, Université de Tours, IFCE, 37380 Nouzilly, France

**Keywords:** reproduction, fertility, oviduct, uterus, endometrium, extracellular vesicles, miRNA, embryo–maternal interactions, implantation, placentation

## Abstract

This Special Issue, “Embryo-Maternal Interactions Underlying Reproduction in Mammals”, gathers a collection of 23 articles, 16 original research articles and 7 up-to-date reviews, providing new findings or summarizing current knowledge on embryo–maternal interactions in seven different mammalian species including humans. Considering the different players involved in these embryo-maternal interactions, articles are mainly focused on one of these different players: the oviduct, the uterus, the embryo or the emergent extracellular vesicles. Additionally, a few articles bring up the impact of reproductive, but also non-reproductive, diseases, as well as stress factors, on the establishment of pregnancy. We hope the readers enjoy this collection of articles and that the knowledge assembled here will support and inspire current and future research investigations. We would like to thank all authors for their contributions to this Special Issue.

## 1. Introduction

**Figure 1:**
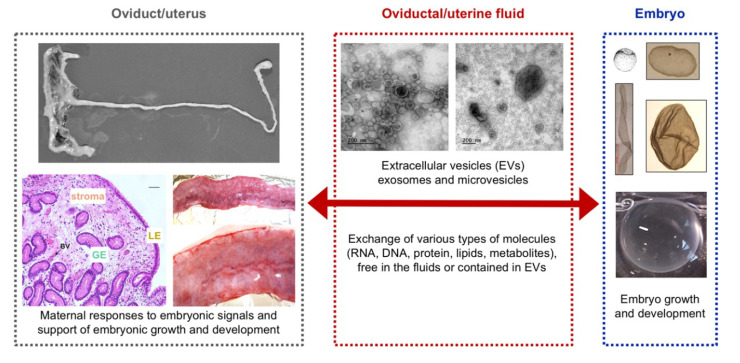
Players in embryo–maternal interactions during the preimplantation phase.

The fascinating diversity in strategies for the recognition and establishment of pregnancy in mammals makes the study of early pregnancy events an exciting field of research. Differences among species have been found regarding the maternal support of embryo development, maternal recognition of pregnancy, preparation for implantation, embryo implantation, and placentation [1,2,3,4,5,6], highlighting the specificity and complexity of these events. The interactions between the embryo(s) and their maternal environment (Figure 1) are considered as the basis of the processes leading to the establishment of successful pregnancy. Different players are involved in these embryo–maternal interactions; on one side the embryo(s), at their different stages of development, and on the other side, the maternal tract including the oviduct and the endometrium, their tissues and fluids surrounding the embryo. More recently, extracellular vesicles/exosomes (EVs) contained in these oviductal and uterine fluids (Figure 1) have emerged as new players in the embryo–maternal dialogue, by modulating the exchange of various types of molecules (RNAs, proteins, lipids, and other metabolites) between them and, also, as an epigenetic mechanism for the transfer of these maternal molecules to the early embryo [7,8,9]. In this Special Issue, seven up-to-date reviews and 16 original research articles gathered current and new knowledge on embryo–maternal interactions in mammals turning to one of the different players: the oviduct, the uterus, the embryo or the EVs. Additionally, a few articles bring up the impact of reproductive, but also non-reproductive, diseases, as well as stress factors, on the establishment of pregnancy.

## 2. The Oviduct

The earliest embryo–maternal interactions are taking place in the oviduct. Oviductal secretions influence from the maturation of gametes and fertilization, to early embryonic development. Two Reviews in this Special Issue summarize current knowledge of the molecular components of the oviductal fluid and their origins, including the factors and processes known to regulate oviductal secretions [10]. Bridi et al. give special emphasis on the role of EVs (oviductal EVs and uterine EVs) in the modulation of embryo–maternal interactions during early pregnancy [7]. The study of Banliat et al. [11] specifically identified a high number of proteins of the bovine oviductal fluid that interact with the early embryo, i.e., 4–8 cell and morula embryos. Changes in the composition of the bovine oviductal fluid isolated from the ampulla and the isthmus were analyzed during the estrous cycle and pregnancy stage in the study by Rodríguez-Alonso et al. [12]. This study revealed that on Day 3 post-estrus, oviductal fluid composition (i.e., differences in amino acids, carbohydrates, and proteins) varied based on the anatomical region of the oviduct and embryo presence, indicating that the composition is anatomically dynamic and affected by the presence of an early embryo [12]. Another study of this Special Issue focused on the identification of proteins present at lower concentrations in the human oviduct and uterus representing the physiological milieu of gamete/embryo–maternal interaction [13]. The rationale of this study was to identify low abundant and specific proteins in the oviductal and uterine fluids which could have a potential role in processes important for embryo–maternal communication and could contribute to the improvement of embryo culture media increasing fertilization rates and early embryo development [13].

## 3. The Uterus

Several studies of the Special Issue focus on alterations of endometrial gene expression during the processes leading to the establishment of pregnancy and the preparation for conceptus implantation in different species. In bovine, endometrial expression of vascular endothelial growth factor A and its receptor FLT1 (VEGFR-1) [14], fibroblast growth factor 1 and 2 (FGF1/2) and their receptors [15], and forkhead box L2 (FOXL2) [16] have been investigated, and roles in early pregnancy and implantation have been suggested. In porcine, the study by Kaczynski et al. [17] compared the effects of the presence of embryos on endometrial gene expression and the main conceptus signal estradiol alone, identifying many differentially expressed genes and processes on day 12 of pregnancy that are regulated by conceptus-derived estrogens, underlining their important role in pregnancy establishment. In equine, an asynchronous embryo transfer model in the mare revealed the extensive adjustment of the conceptus transcriptome in response to a negatively asynchronous uterus, whereas the endometrial transcriptome changes were only subtly due to the presence of a more advanced conceptus. The analysis of the endometrium and conceptus transcriptome in this study revealed a variety of processes and genes important to the establishment of pregnancy in equids [18].

Two Reviews are specifically focusing on the role of microRNAs in establishment and maintenance of pregnancy. Salilew-Wondim et al. highlight the role of miRNAs in mammalian gametogenesis and embryogenesis [19], while Kaczmarek et al., focus on their contribution to the embryo–maternal communication, particularly in pigs [20]. Furthermore, two original articles provide new clues about the roles of miRNAs in embryo–maternal communication in the horse. Smits and co-workers [21] performed an integrated analysis of differential miRNA, mRNA, and protein expression analysis at the embryo–maternal interface. In agreement with previous studies [22,23], the results derived from their study did not point at of an unequivocal signal for MRP in the horse but highlighted a potential role of miRNAs in embryo–maternal communication during pregnancy establishment in the horse. The second article studied the expression of the chromosome 14 microRNA cluster (C14MC) in the maternal circulation throughout pregnancy [24]. This study revealed dynamic profile of miRNA over time and also between pregnant and cyclic mares, pointing at serum eca-miR-1247-3p, eca-miR-134-5p, and eca-miR-409-3p expression as pregnancy-specific markers during early gestation.

In addition to maternal recognition of pregnancy, the processes of implantation and placentation are very critical. Ochoa-Bernal and Fazleabas summarize the current knowledge of the cellular and molecular mechanisms controlling these processes in women and non-human primates [5]. Moreover, these authors point out possible pathologies related to defects in implantation and decidualization. In sheep, the study of Seo et al. revealed that the uterine luminal epithelial cells are not incorporated into the syncytial plaques formed during early placentation, in contrast to previous findings [25]. This finding suggest that early placentation in sheep is similar to early placentation in humans, since both develop mononucleated cytotrophoblast and multinucleated syncytiotrophoblast layers of entirely placental origin [25].

## 4. The Embryo

Since embryo–maternal communication is a two-way interaction, the study of embryo-derived signals is an essential part to be considered as well. The review by Fujiwara et al. highlights the roles of embryonic signals during implantation and placentation, and, particularly, their effects on endocrine and immune systems [6]. Despite that the major pregnancy recognition signal in ruminants, interferon-tau, has been discovered long time ago [26], more and more studies are showing that other embryonic molecules are involved in the establishment of pregnancy, pointing out the high complexity of these interactions. In this line, Malo Estepa et al., examined the protein synthesis by day 16 conceptuses corresponding to the phase of maternal recognition of pregnancy [27], identifying a set of conceptus-derived proteins that may be involved in EV-mediated IFNT-independent embryo–maternal communication during pregnancy recognition in cattle.

## 5. The Extracellular Vesicles

An increasing number of studies are pointing at the EVs as key players in the regulation of embryo–maternal interactions [7,8,9,10]. Thus, the study of the EVs molecular cargo at RNAs, protein and lipid levels aiming at revealing clues about their potential roles in these interactions has gained special attention in recent years. Bridi et al. summarize the current knowledge about the potential roles of both maternal (including oviduct and uterus) and embryonic EVs in the crucial events leading to successful pregnancy [7]. To increase the current knowledge of the oviductal EVs molecular cargo, Gatien et al. focused on a less known cargo, characterizing metabolomic profile of bovine oviductal EVs during the estrous cycle [28]. Their study revealed up to 100-fold higher levels of glucose-1-phosphate and maltose at the luteal phase compared to the peri-ovulatory phases. Furthermore, to evidence the functional impact of oviductal EVs on the early embryos, the effects of the oviductal EVs molecular cargo on the transcriptome of bovine in vitro produced embryos were investigated [29]. The integrative analysis of mRNAs and miRNAs identified in oviductal EVs in a previous study [30], with the observed mRNA alterations in the embryos after oviductal EVs co-incubation for a week, revealed direct effects of the oviductal EV cargo on the embryo. The oviductal EVs cargo might act by increasing the concentration of delivered transcripts, translation of delivered mRNAs into functional proteins, and/or via oEV-derived miRNAs.

## 6. The Impact of the Diseases and Stress on the Establishment of Pregnancy.

Three papers of the Special Issue focus on how reproductive but also non-reproductive diseases affect the establishment of pregnancy. Canisso et al. address clinical aspects of persistent breeding-induced endometritis in the mare, the mechanisms of the pathogenesis, and discuss currently available and potential future therapies [31]. Schindler et al. analyzed the metabolic profiling of blood plasma and blastocoel fluid of diabetic rabbits, revealing profound effects of the diabetes on both the maternal metabolism and the metabolism of the embryo as reflected by the changes in blastocoel fluid metabolites [32]. Considering that stress is also a negative factor affecting fertility, the study of Du et al. revealed the effects of elevated cortisol levels on the early embryonic environment using a porcine oviduct epithelium in vitro model [33].

## 7. Conclusions

This Special Issue represents a collection of 23 original and up-to-date review articles, providing new findings or summarizing current knowledge on embryo–maternal interactions underlying reproduction in seven different mammalian species including humans. The studies gathered here clearly show how omics technologies at the level of transcripts, proteins, and metabolites have revolutionized the study of embryo–maternal interactions during the establishment of pregnancy. Moreover, their use has revealed an unexpected complexity and diversity of the cellular and molecular embryo–maternal dialog, contributing to the beginning of a new life in mammals.

The current challenge is to use this knowledge to enhance fertility and reproductive health in humans and animals. Moreover, considering the impact of the exposome of today´s society, including stressful environments or common diseases as diabetes or obesity, on the reproductive success, the complexity of known and unknown mechanisms and involved molecules is even higher. Therefore, we believe that reproductive research should be a main focus in academic and non-academic research as well as in future strategies of funding bodies. Successful reproduction is essential for the future of all of us; humans, domestic and wild animals.

Finally, we would like to thank all authors for their contributions to this Special Issue. We hope the readers enjoy this collection of articles and that the knowledge assembled here help with their current and future research investigations.
